# Placental DAPK1 and autophagy marker LC3B-II are dysregulated by TNF-α in a gestational age-dependent manner

**DOI:** 10.1007/s00418-016-1537-1

**Published:** 2017-01-17

**Authors:** Andreas Prokesch, Astrid Blaschitz, Tamara Bauer, Gerit Moser, Ursula Hiden, Julianna Zadora, Ralf Dechend, Florian Herse, Martin Gauster

**Affiliations:** 1 Institute of Cell Biology, Histology and Embryology, Medical University Graz, Harrachgasse 21/VII, 8010 Graz, Austria; 2 Department of Obstetrics and Gynaecology, Medical University Graz, Graz, Austria; 3 Experimental and Clinical Research Center, A Joint Cooperation Between the Charité Medical Faculty and the Max-Delbrueck Center for Molecular Medicine, Berlin, Germany; 4 Berlin Institute of Health (BIH), Berlin, Germany; 5 HELIOS-Klinikum Berlin, Berlin, Germany

**Keywords:** Pregnancy, Preeclampsia, Placenta, Trophoblast, Inflammation, Autophagy

## Abstract

Autophagy, a cell-survival process responsible for degradation of protein aggregates and damaged organelles, is increasingly recognized as another mechanism essential for human placentation. A substantial body of experiments suggests inflammation and oxidative stress as the underlying stimuli for altered placental autophagy, giving rise to placenta dysfunction and pregnancy pathologies. Here, the hypothesis is tested whether or not pro-inflammatory cytokines interleukin (IL)-6 and tumor necrosis factor (TNF)-α are able to influence the expression profile of autophagy genes in human first-trimester villous placenta. Autophagy-focused qPCR arrays identified substantial downregulation of death-associated protein kinase 1 (DAPK1) in first-trimester placental explants in response to IL-6 and TNF-α, respectively. Immunohistochemistry of placental explants detected considerable DAPK1 staining in placental macrophages, villous cytotrophoblasts and less intense in the syncytiotrophoblast. Both immunohistochemistry and Western blot showed decreased DAPK1 protein in TNF-α-treated placental explants compared to control. On cellular level, DAPK1 expression decreased in SGHPL-4 trophoblasts in response to TNF-α. Observed changes in the expression profile of autophagy-related genes were reflected by significantly decreased lipidation of autophagy marker microtubule-associated protein light chain 3 beta (LC3B-II) in first trimester placental explants in response to TNF-α. Analysis of TNF-α-treated term placental explants showed decreased DAPK1 protein, whereas in contrast to first-trimester LC3B expression and lipidation increased. Immunohistochemistry of placental tissues from early-onset preeclampsia (PE) showed less DAPK1 staining, when compared to controls. Accordingly, DAPK1 mRNA and protein were decreased in primary trophoblasts isolated from early-onset PE, while LC3B-I and -II were increased. Results from this study suggest that DAPK1, a regulator of apoptosis, autophagy and programmed necrosis, decreases in human placenta in response to elevated maternal TNF-α, irrespective of gestational age. In contrast, TNF-α differentially regulates levels of autophagy marker LC3B in human placenta over gestation.

## Introduction

Concerted placenta development is mandatory for successful human pregnancy and depends on a fine balanced cross-talk of immune-modulating factors between fetal and maternal tissues. Amongst immune-modulating factors, pro- and anti-inflammatory cytokines play a critical role in regulating cellular processes such as cell proliferation, differentiation as well as apoptosis to overall assure proper placental growth and development in particular during very early phase of human pregnancy. At that stage, macroautophagy (herein referred to as autophagy)—a cell-survival process responsible for degradation of aggregates of misfolded proteins or damaged organelles—is increasingly recognized as essential to maintain a cellular balance between anabolic and catabolic processes, allowing appropriate growth of the embryo and placenta (Kanninen et al. 2013). In normal gestation autophagy becomes activated early in human placenta and can be detected in placental key cells including cytotrophoblast, syncytiotrophoblast, and extravillous trophoblast, as well as maternal decidual stroma cells (Avagliano et al. 2015). Hence, autophagy is considered as cytoprotective response to different stress signals, including starvation, pathogens, hypoxia, reactive oxygen species and cytokines (Schmeisser et al. 2014). For human placenta, even the mode of delivery seems to influence autophagic activity, with higher levels of autophagy markers in placentas from cesarean section compared to vaginal delivery (Signorelli et al. 2011). A substantial body of in vitro experiments using primary trophoblasts and cell lines suggests inflammation and oxidative stress as the underlying stimuli for altered placental autophagy, giving rise to pregnancy pathologies, such as preeclampsia (Akaishi et al. 2014; Kalkat et al. 2013; Oh et al. 2008) and fetal growth restriction (Curtis et al. 2013). On the basis of mRNA and protein levels of autophagy markers, such as the posttranslationally modified active form of microtubule-associated protein 1 light chain 3 beta (LC3B-II), lysosome-associated membrane protein 2 (LAMP-2) and beclin-1, increased autophagy is described in placentas from pregnancies complicated by fetal growth restriction, with or without preeclampsia, but not for those with pre-eclampsia alone (Curtis et al. 2013; Hung et al. 2012). In contrast, other studies showed evidence for increased autophagy in placentas from hypertensive pregnancies, independent of the presence of growth restriction (Akaishi et al. 2014; Oh et al. 2008). Besides hypertension, maternal body mass index (BMI) and fetal sex seem to influence placental autophagy, as activation of autophagosomal formation and autophagosome–lysosome fusion have been shown in placentas from male but not female offspring of overweight and obese women. However, total autophagic activity in these placentas appears to be decreased as evidenced by an increase in p62 and a decrease in lysosomal biogenesis (Muralimanoharan et al. 2016). Of note, obesity is accompanied by enhanced inflammatory conditions, with increased TNF-α and IL-6 levels (Gregor and Hotamisligil 2011).

In preterm delivery cases with histological signs of inflammation, placental expression of autophagy marker LC3 is associated with the severity of fetal inflammation and is significantly decreased compared to age-matched controls (Avagliano et al. 2016). Overall, a moderate to strong pro-inflammatory maternal environment in these pregnancy complications may be responsible for altered placental autophagy. Indeed, pro-inflammatory T helper 1 (Th1) cytokines such as interleukin (IL)-1, IL-2, IL-6, tumor necrosis factor (TNF)-α, transforming growth factor (TGF)-β and interferon (IFN)-γ have been demonstrated to induce autophagy, whereas anti-inflammatory Th2 cytokines IL-4 and IL-13 inhibit it (Harris 2011). However, it is increasingly acknowledged that metabolic and pro-inflammatory conditions influence early placenta development and functions in the first trimester of pregnancy long before any phenotypic changes become clinically apparent (Catalano and deMouzon 2015). Dysregulation of the autophagy machinery in response to a pro-inflammatory environment may contribute to aberrant placenta development and dysfunction. Thus, the question addressed was whether exogenously applied IL-6 and TNF-α, mimicking increased maternal pro-inflammatory cytokines, are able to influence the autophagy program on transcriptional level in human first-trimester villous placenta. Moreover, observed effects of TNF-α on autophagy markers in human first-trimester placenta were compared to term placenta and early-onset preeclampsia cases.

## Materials and methods

### Human placenta tissue samples

The study was approved by the ethical committee of the Medical University of Graz. First-trimester placenta tissues (*n* = 8, mean gestational week: 10.6 ± 1.8) were obtained with informed written consent from healthy women (mean maternal age: 26.0 ± 6.9 years; mean body mass index: 21.2 ± 2.6) undergoing elective pregnancy terminations for psychosocial reasons. Term placentas for placental explant culture were obtained immediately after caesarean section (*n* = 3, mean gestational week: 39.7 ± 0.6) from healthy women with singleton pregnancies.

### Placental explant culture

For placental explant culture villous tissues from human first-trimester and term placentas were washed thoroughly in buffered saline and dissected into small pieces of approximately 5 mg moist mass. Placental explants were cultured in 12-well dishes (nunc, Thermo Scientific) and 2mL/well DMEM/F12 (1:1, Gibco) supplemented with 10% FCS, penicillin/streptomycin, amphotericin B, and L-glutamine in a hypoxic workstation (BioSpherix) under 2.5% oxygen (for first trimester) and 8% oxygen (for term), respectively, at 37 °C for 48h. For cytokine treatments, complete culture medium was supplemented with 10 ng/ml recombinant human IL-6 (Peprotech) and recombinant human TNF-α (Peprotech), respectively. Cultivation of explants in complete culture medium without cytokines served as controls. After incubation, placental explants were homogenized for RNA isolation using peqGOLD TriFast reagent (Peqlab, Erlangen, Germany) and a tissue homogenizer (IKA TA10 basic, Ultra-Turrax). A proportion of placental explants cultured in the same experimental setup were either formalin fixed and paraffin embedded (FFPE) for subsequent immunohistochemistry or homogenized in RIPA buffer (Sigma-Aldrich; St. Louis, MO, USA) including Protease Inhibitor Cocktail (Roche Diagnostics; Mannheim, Germany) for Western blot analyses. Homogenates were centrifuged at 8000×*g* and 4 °C for 10 min. Concentration of total tissue protein was determined in homogenates according to Lowry method.

### Analysis of placental explant viability

Potential cytotoxic effects of IL-6 and TNF-α on placental explants were evaluated by measurement of released lactate dehydrogenase (LDH) activity in culture supernatants using LDH Cytotoxicity Detection Kit (Takara Bio Inc., eubio; Vienna; Austria) according to the manufacturer’s protocol. Obtained absorbance values were normalized to total protein of respective explant homogenates.

### Isolation of primary trophoblasts from human placenta

Human placenta sampling was approved by the Regional Committee of the Medical Faculty of Charité Berlin. Primary trophoblasts were isolated from chorionic villi by enzymatic digestion and Percoll density gradient centrifugation as previously described (Przybyl et al. 2016). Primary trophoblasts were isolated from placentas of uncomplicated pregnancies collected following caesarean section at term (control trophoblasts, *n* = 6) and from placentas of pregnancies complicated by early-onset preeclampsia (*n* = 5). Early-onset preeclampsia was defined by hypertension (SBP ≥ 140 mmHg or DBP ≥ 90 mmHg) and proteinuria (≥0.3 g in a 24-h urine specimen) before 34th gestational week according to the American College of Obstetricians and Gynecologists (ACOG) classification.

### Cell culture

Trophoblast cell line SGHPL-4 was a kind gift from Judith E. Cartwright (St. George’s University of London, London, United Kingdom) and was cultivated in HAM’s F10 (Biochrom) media containing 10% (v/v) FCS and 1% (v/v) P/S. For expression analysis, starved SGHPL-4 cells were stimulated with 10 ng/ml TNF-α (Sigma-Aldrich). Total mRNA was isolated at indicated time points using QIAzol lysis reagent and Qiagen RNeasy mini-kit (Qiagen) with on-column deoxyribonuclease I step (Qiagen) according to the manufacturer’s protocol.

### Gene expression analysis

Total RNA from placental tissue was isolated with peqGOLD TriFast reagent according to the manufacturer’s protocol. For human autophagy PCR array, RNA was subjected to quality check and cDNA synthesis using the RT^2^ First Strand Kit (Qiagen) according to the manufacturer’s manual. In brief, total RNA from explants of each treatment was pooled and 1 µg was used for genomic DNA elimination and reverse transcription. cDNA was used for the real-time Human Autophagy RT² Profiler PCR Array (PAHS-084Z, Qiagen) in combination with RT² SYBR Green qPCR Mastermix (Qiagen) on a Bio-Rad CFX96 Real-Time PCR System. cDNA of each treatment was run on a single plate and automatically generated *C*_t_ values were analyzed using the data analysis web portal http://www.qiagen.com/geneglobe. All samples passed PCR array reproducibility, RT efficiency and genomic DNA elimination. Fold change was calculated using ΔΔ*C*_t_ method, in which Δ*C*_t_ was calculated between gene of interest and arithmetic mean of two normalization genes hypoxanthine–guanine phosphoribosyltransferase (HPRT1) and ribosomal protein P0 (RPLP0).

Results from human autophagy PCR array were evaluated for selected genes by probe-based qPCR. For this purpose, 2 µg of total RNA from each sample was individually reverse transcribed to cDNA, using High-Capacity cDNA Reverse Transcription Kit (Applied Biosystems; Foster City, CA, USA). cDNA was subsequently subjected to qPCR using TaqMan Gene Expression Assays for Cathepsin D (CTSD, Hs00157205_m1), Death-associated protein kinase 1 (DAPK1, Hs00234480_m1), and Histone deacetylase 6 (HDAC6, Hs00195869_m1), and the TaqMan Universal PCR Mastermix (Applied Biosystems). cDNA and kit components were mixed in 20 µl total volume/well (96-well plates, Roche, Mannheim, Germany) according to the manufacturer’s instructions and amplified using a Bio-Rad CFX96 Real-Time PCR System. Expression of LC3B was analyzed as previously described (Prokesch et al. 2016) using SYBR Green chemistry (Bio-Rad, Hercules, CA, USA) and specific primers for MAP1LC3B (MAP1LC3B_For: AAGGCGCTTACAGCTCAATG and MAP1LC3B_Rev: CTGGGAGGCATAGACCATGT). *C*_t_ values were automatically generated by the CFX Manager 2.0 Software (Bio-Rad) and relative quantification of gene expression was calculated by standard Δ*C*_t_ method using the expression of RPL30 and 18S as references. Data are presented as mean of 2^−Δ*C*t^ values. Gene expression in SGHPL-4 trophoblasts was analyzed on ABI 7500 Fast Sequence Detection System (Applied Biosystems) and analyzed by 7500 Fast System Software (Applied Biosystems).

### Immunohistochemistry

FFPE placental sections (5 μm) were mounted on Superfrost Plus slides (Menzel/Thermo Fisher Scientific) and deparaffinized according to the standard protocol. Slides were boiled in epitope retrieval solution pH 8.0 (Novocastra, Leica) for 7 min at 120 °C using a decloaking chamber (Biocare Medical). Immunostaining was performed with the UltraVision Detection System HRP Polymer Kit (Thermo Fisher Scientific) as previously described (Blaschitz et al. 2015; Siwetz et al. 2015). In brief, endogenous peroxidase was blocked using the hydrogen peroxidase block for 10 min. Three washing steps with TBS including 0.05% Tween 20 (TBS/T; Merck) were followed by background blocking with UltraVision Protein Block for 5 min. Polyclonal rabbit anti-DAPK1 antibody (ab200549, abcam) was diluted to a final concentration of 1 µg/ml in Antibody Diluent (Dako) and incubated on slides for 45 min at RT. Thereafter slides were washed three times and detection was achieved by incubation with anti-rabbit UltraVision HRP-labelled polymer (15 min) and 3-amino-9-ethylcarbacole (AEC, Thermo Scientific) for 10 min. Nuclei were stained with hemalaun and slides were mounted with aqueous mounting agent Aquatex (Merk Millipore). For negative control, slides were incubated with negative control for rabbit IgG (Neomarkers, ThermoScientific, USA) at the same concentration as for anti-DAPK1 antibody.

### Western blot analysis

Placental explants were washed with PBS after culture and homogenized in RIPA buffer including protease inhibitor cocktail. Primary trophoblasts were lysed in RIPA buffer after cell isolation procedure. Protein concentration was determined according to Lowry method and 40 µg of total protein was separated on precast 10% Bis-Tris gels (NuPAGE, Novex; lifetechnologies). Electrophoresis was followed by semi-dry blotting of proteins on 0.45-µm nitrocellulose membrane (Hybond, GE Healthcare Life Sciences) and blotting efficiency was determined by staining membranes with Ponceau S solution (Sigma-Aldrich). Immunodetection was performed with a chemiluminescent immunodetection kit (Amersham ECL Prime Western blotting detection Reagent, GE Healthcare Life Sciences) according to the manufacturer’s instructions. Membranes were cut into horizontal strips at molecular weight ranges for target proteins. Polyclonal rabbit anti-DAPK1 antibody (1:1000, ab200549, abcam), polyclonal LC3B/MAP1LC3B antibody (1:1000, NB100-2220, novusbio) and monoclonal anti-beta actin antibody (1:20,000; clone AC-15, abcam) were diluted in blocking solution and membrane strips were incubated overnight at 4 °C. Images were acquired with FluorChem Q System (Alpha Innotech, Cell Bioscienes, Santa Clara, CA, USA) and band densities were analyzed with Alpha View SA software 3.4.0. Results are presented as a ratio of relative DAPK1 or LC3B and beta-actin band densities.

### Statistical analysis

Data were analyzed using GraphPad Prism 5.01 for Windows (GraphPad Software, San Diego California USA, http://www.graphpad.com) and are presented as means ± SEM. Data were subjected to D’Agostino and Pearson omnibus normality test. In case of normally distributed data, differences between groups were tested using paired two-tailed *t* test. For multiple comparison procedure, one-way repeated measures analysis of variance was followed by Dunnett’s multiple comparison test to isolate groups that differ from control. *p* < 0.05 was considered statistically significant.

## Results

**Fig. 1:**
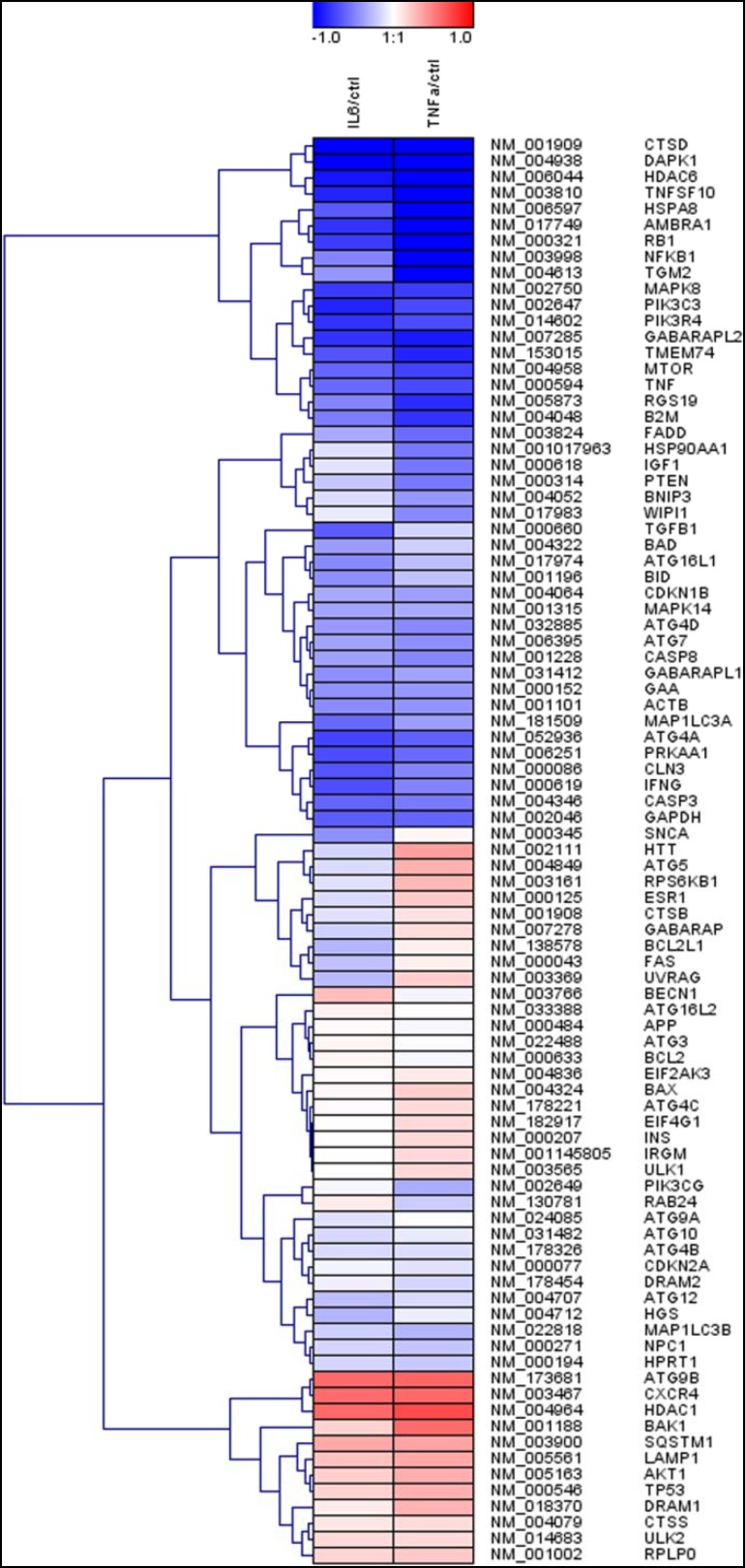
Hierarchical clustering of autophagy-related genes in IL-6- and TNF-α-treated placental explants. Human first-trimester placental explants from different patients (*n* = 8) were incubated with or without IL-6 (10 ng/ml) and TNF-α (10 ng/ml), respectively, for 48h. RNA from each treatment group was pooled and subjected to autophagy qPCR array. The heat map with dendrograms shows log2 fold-changes in blue for downregulated and in red for upregulated autophagy-related genes in IL-6-treated (*left column*) and TNF-α- treated (*right column*) placental explants relative to untreated controls

To determine effects of an inflammatory microenvironment on the placental gene expression profile of genes involved in autophagy early in gestation, human placental explants from first trimester of pregnancy were incubated in the presence or absence of pro-inflammatory cytokines IL-6 and TNF-α, respectively. Analysis of LDH release into the culture medium did not show significant differences between treatments, suggesting no cytotoxic effects of cytokines at used concentrations (data not shown). However, analysis of expression profile of 84 autophagy-related genes in first-trimester placental explants showed a similar shift in the presence of IL-6 and TNF-α after 48h culture. Comparison of normalized expression of every gene on the array between cytokine-treated explants and controls indicated a higher proportion of downregulated than upregulated genes (Fig. 1). Amongst the array of analyzed genes 78.1 and 63.5% were downregulated in the presence of IL-6 and TNF-α, respectively. Notably, in both IL-6- and TNF-α-treated first-trimester placental explants, autophagy-related 9B (ATG9B), C-X-C chemokine receptor type 4 (CXCR4), and histone deacetylase 1 (HDAC1) were the three most upregulated genes, ranging from 1.49- to 1.63-fold upregulation, when compared to control. In contrast, IL-6 treatment induced 2.08- and 2.05-fold downregulation of cathepsin D (CTSD) and death-associated protein kinase 1 (DAPK1), respectively. In TNF-α-treated first-trimester placental explants, nine autophagy-related genes were more than twofold downregulated, including, CTSD (−2.17-fold), heat-shock 70-kDa protein 8 (HSPA8, −2.27-fold), DAPK1 (−2.34-fold), nuclear factor of kappa light polypeptide gene enhancer in B cells 1 (NFKB1, −2.35-fold), tumor necrosis factor superfamily, member 10 (TNFSF10, −2.40-fold), and histone deacetylase 6 (HDAC6, −2.56-fold), as the most downregulated. Interestingly, unsupervised hierarchical clustering of the entire array dataset indicated similar regulatory effects on autophagy-related genes by IL-6 and TNF-α, respectively (Fig. 1).

**Fig. 2:**
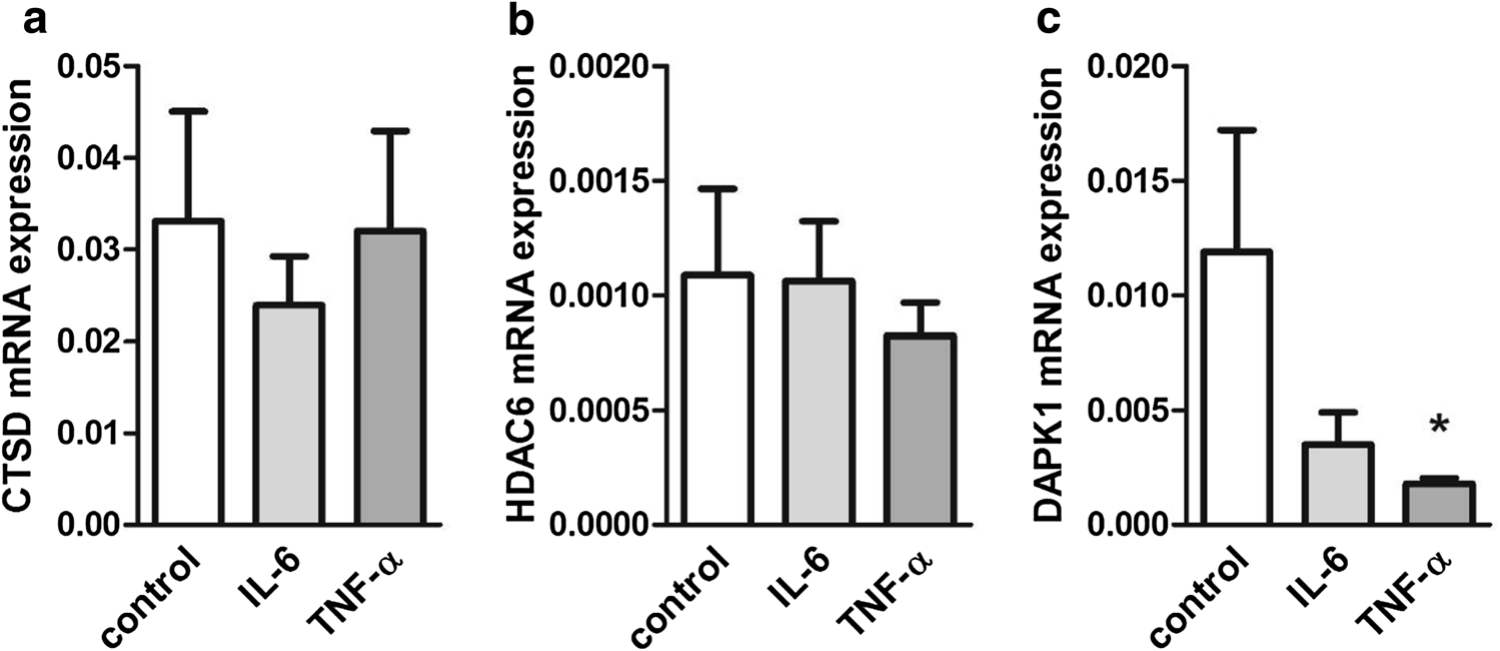
Expression analysis of CTSD, HDAC6, and DAPK1 in IL-6- and TNF-α-treated placental explants. Human first-trimester placental explants (*n* = 8) were incubated with or without IL-6 (10 ng/ml) and TNF-α (10 ng/ml), respectively, for 48h. qPCR analysis was performed for CTSD (**a**), HDAC6 (**b**), and DAPK1 (**c**). Values are given as mean ± SEM. *p < 0.05

Next, results from autophagy PCR array were evaluated in individual first-trimester explant samples for three top-ranked downregulated genes (CTSD, DAPK1, and HDAC6) by qPCR analysis using a different set of primers. CTSD (−1.38- and −1.04-fold) and HDAC6 expression (−1.03- and −1.33-fold) were slightly, but not significantly downregulated in the presence of IL-6 and TNF-α, respectively (Fig. 2a, b). However, DAPK1 showed a substantial 3.40- and significant 6.58-fold (*p* < 0.05) downregulation by IL-6 and TNF-α, respectively (Fig. 2c). Since effects of IL-6 did not reach statistical significance, further experiments were performed only with or without TNF-α treatments.

**Fig. 3:**
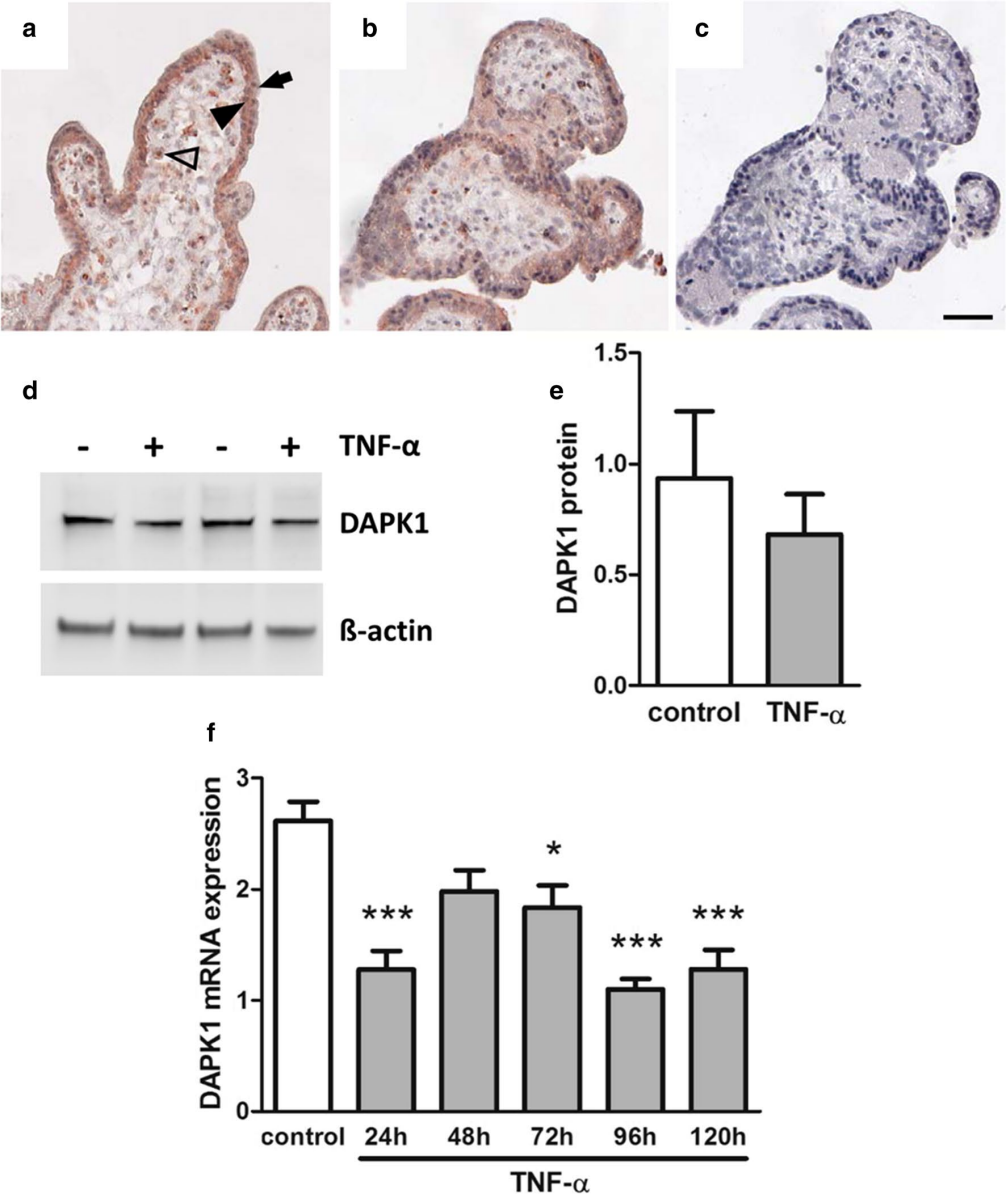
DAPK1 protein expression in TNF-α treated placental explants. Human first trimester placental explants were either treated in the absence (**a**) or presence (**b**) of TNF-α (10 ng/ml) for 48h. Immunohistochemistry detected DAPK1 in Hofbauer cells (*open arrowhead*), villous cytotrophoblasts (*black arrowhead*) and with less intensity in the syncytiotrophoblast (*arrow*). Incubation with negative control rabbit IgG (**c**) revealed no staining. *Scale bar* represents 50 µm. Western blot analysis (**d**) of placental explants treated with or without TNF α (10 ng/ml) for 48h and band densitometry of placental DAPK1 levels (**e**) in response to cytokine treatment. Band densities of DAPK1 were related to corresponding densities of β-actin. Data are presented as mean ± SEM from five different explant cultures. Trophoblast cell line SGHPL-4 was treated with or without TNF α (10 ng/ml) for up to 120 h and DAPK1 expression was analyzed by qPCR (**f**). Data are presented as mean ± SEM from three independent experiments. *p < 0.05, ***p < 0.001

Immunohistochemistry of first-trimester placental explants detected marked DAPK1 staining in placental macrophages (Hofbauer cells) and the villous trophoblast layer, where cytotrophoblasts showed more intense staining than the syncytiotrophoblast (Fig. 3a). TNF-α-treated first-trimester placental explants showed a similar, but less pronounced staining of villous trophoblasts compared with controls (Fig. 3b). Western blot analysis of first-trimester placental explants detected DAPK1 protein at a molecular weight of approximately 160 kDa (Fig. 3d). Placental DAPK1 protein levels decreased by 27.1% in response to TNF-α, which, however, did not reach statistical significance (Fig. 3e). Accordingly, on cellular level, DAPK1 mRNA expression significantly decreased in first-trimester trophoblast cell line SGHPL-4 during a 120-h TNF-α treatment (Fig. 3f).

**Fig. 4:**
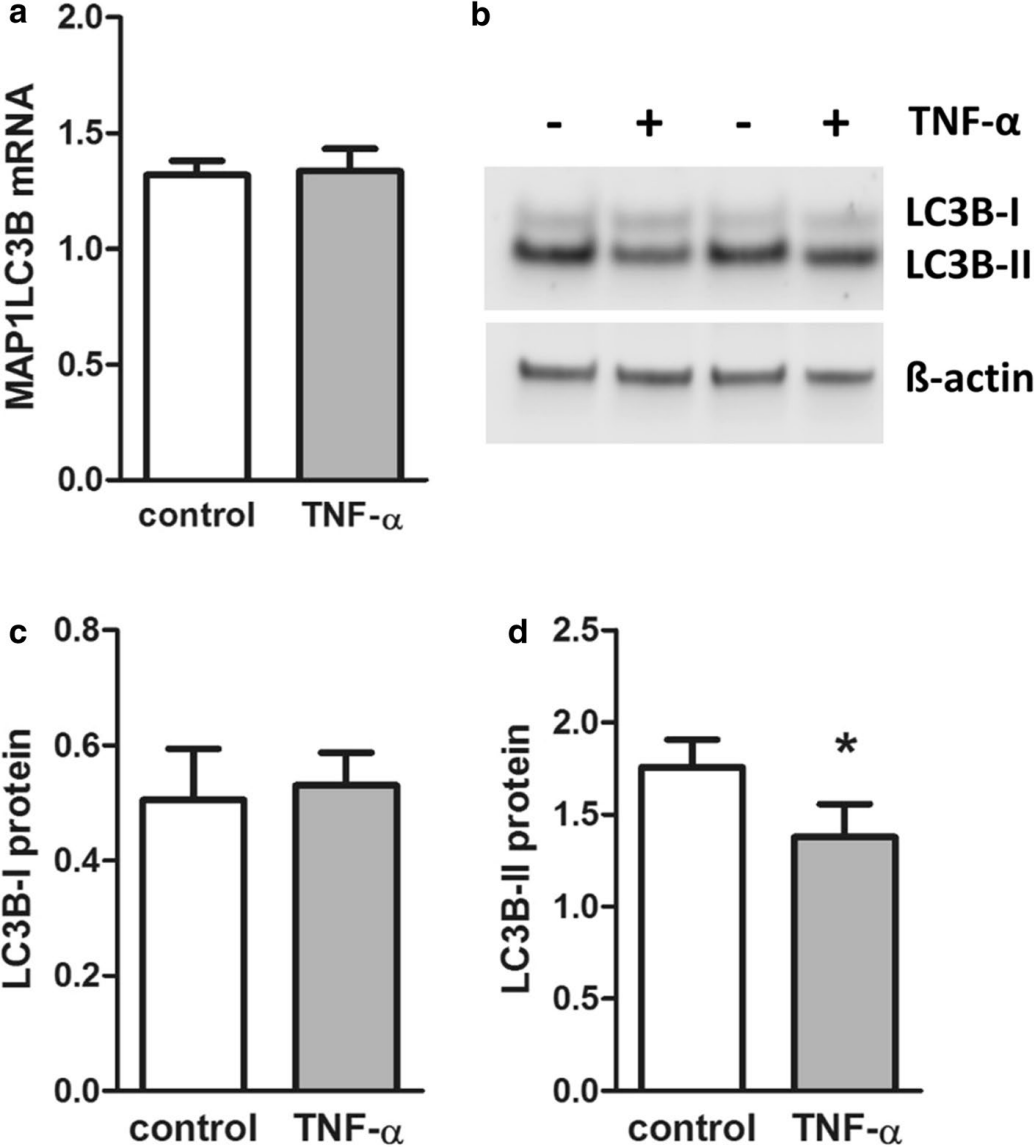
LC3B levels in TNF-α-treated placental explants. Human first-trimester placental explants were incubated with or without TNF-α (10 ng/ml) for 48h. qPCR analysis of LC3B gene expression (**a**), Western blot analysis (**b**) and band densitometry data for LC3B-I (**c**) and LC3B-II (**d**), respectively, which were related to corresponding densities of β-actin. Data are presented as mean ± SEM from eight (**a**) and five (**b–d**) different explant cultures. *p < 0.05

To test whether or not cytokine-induced changes in the expression profile of autophagy-related genes were reflected by altered placental autophagy, effects of TNF-α on autophagy marker LC3B were analyzed in first-trimester placental explants. qPCR analysis of the LC3B gene MAP1LC3B did not show regulation by TNF-α on mRNA expression (Fig. 4a), confirming data from autophagy array. On protein level, LC3B appears in two variants: as unmodified, cytosolic variant (LC3B-I) and the posttranslationally processed LC3B-II, which undergoes lipidation with phosphatidylethanolamine and binds to the outer membrane of autophagosomes. Increase in LC3B-II is, therefore, a marker of activated autophagy (Klionsky et al. 2016). Accordingly, Western blot analysis of first-trimester placental explants for LC3B detected two bands at approximately 19 and 17 kDa corresponding to LC3B-I and LC3B-II, respectively. Interestingly, levels of placental LC3B-II were consistently stronger than LC3B-I, irrespective of the presence or absence of TNF-α (Fig. 4b). However, while levels of LC3B-I remained unchanged, incubation with TNF-α decreased placental LC3B-II levels by 21.5% (*p* = 0.025) when compared to controls (Fig. 4b–d). This TNF-α-mediated reduction in autophagy is consistent with the predominant downregulation of autophagy-related genes, such as DAPK1.

**Fig. 5:**
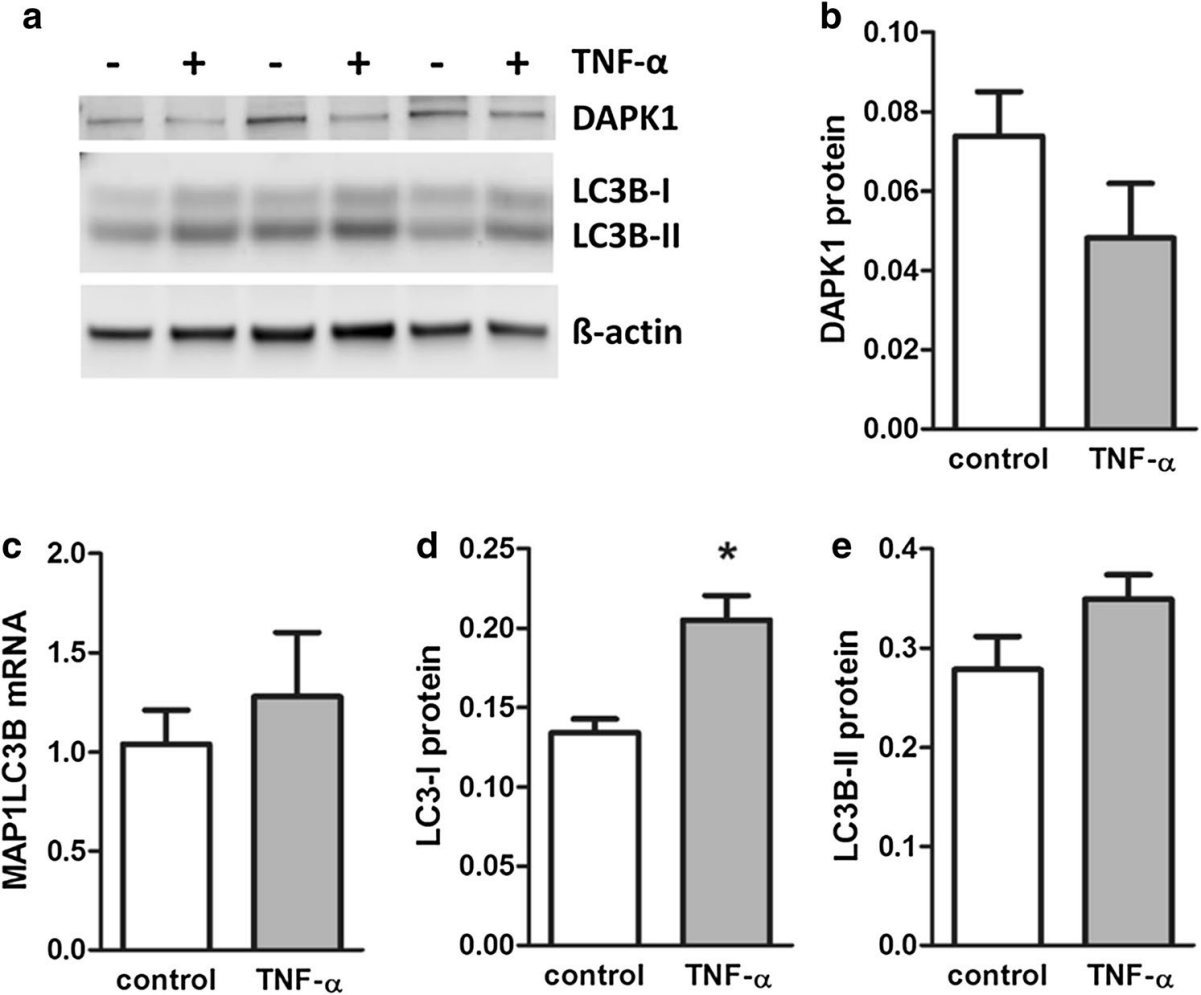
DAPK1 and LC3B levels in TNF-α treated term placental explants. Human term placental explants were incubated with or without TNF-α (10 ng/ml) for 48h. Western blot analysis of DAPK1 and LC3B isoforms (**a**) and band densitometry data for DAPK1 protein (**b**), qPCR analysis of LC3B gene expression (**c**), as well as band densitometry data for LC3B isoforms LC3B-I (**d**) and LC3B-II (**e**), respectively, which were related to corresponding densities of β-actin. Data are presented as mean ± SEM from three different explant cultures. *p < 0.05

Next we tested whether or not effects observed for first trimester also apply for term placenta tissue. For this purpose, term placental explants were incubated in the presence or absence of TNF-α. While qPCR analysis revealed no change on DAPK1 mRNA levels (data not shown), DAPK1 protein levels decreased by 34.8% in response to TNF-α, which, however, did not reach statistical significance (Fig. 5a, b). Analysis of autophagy marker LC3B in term placental explants showed a non-significant 1.23-fold increase in MAP1LC3B mRNA expression in response to TNF-α (Fig. 5c). On protein level, LC3B protein showed a similar picture as in first trimester, with consistently stronger LC3B-II than LC3B-I levels, irrespective of treatment (Fig. 5a). However, in contrast to first trimester, both LC3B-I (p = 0.016) and LC3B-II levels increased in term placental explants in response to TNF-α treatment (Fig. 5d, e).

**Fig. 6:**
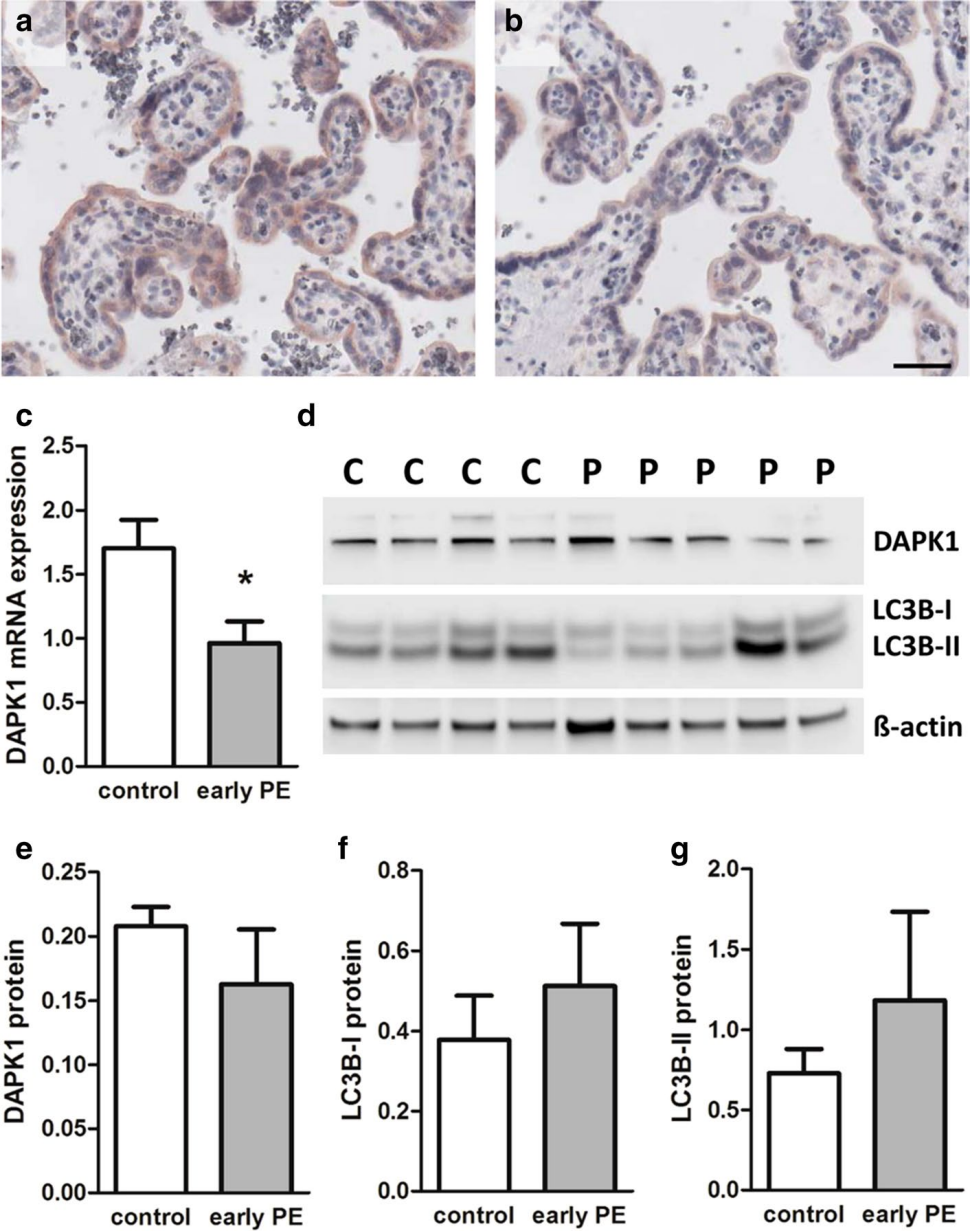
DAPK1 expression and LC3B levels in trophoblast and placentas from pregnancies complicated by preeclampsia. Immunohistochemistry for DAPK1 in placental tissue sections from control (**a**) and early-onset preeclampsia (**b**). *Scale bar* represents 50 µm. DAPK1 expression was analyzed by qPCR in primary trophoblasts isolated from control placentas (*n* = 6) and placentas from early-onset preeclampsia (early PE, *n* = 5) (**c**). Western blot analysis (**d**) and band densitometry data for DAPK1 protein (**e**), and LC3B isoforms LC3B-I (**f**) and LC3B-II (**g**), respectively, in primary trophoblasts isolated from control (*n* = 6) and early PE (*n* = 5) placentas. Band densities of target proteins were related to corresponding densities of β-actin. Data are presented as mean ± SEM. *p < 0.05

Finally, DAPK1 expression and autophagy marker LC3B were analyzed in placental tissue and primary trophoblasts isolated from placentas complicated by early-onset preeclampsia (PE), a condition associated with increased maternal circulating pro-inflammatory cytokines (Lau et al. 2013). Immunohistochemistry of placental tissues from control (Fig. 6a) and early-onset PE (Fig. 6b) localized DAPK1 in the syncytiotrophoblast, which was less stained in early-onset PE cases, compared to controls. Consistently, primary trophoblasts isolated from placentas complicated by early-onset PE showed a 1.77-fold (*p* < 0.05) decrease in DAPK1 mRNA expression, when compared to trophoblasts isolated from healthy control placentas (Fig. 6c). On protein level, both DAPK1 and LC3B showed strong within-group variations, in both controls and PE cases. However, downregulation of DAPK1 was confirmed by trend, showing a decrease by 21.6% in trophoblasts from early-onset PE (Fig. 6d, e). Analysis of LC3B levels in these samples revealed a 1.36- and 1.62-fold increase in LC3B-I and LC3B-II levels, respectively, in trophoblasts isolated from preeclamptic placentas, when compared to controls (Fig. 6d, f, g).

## Discussion

Here we report the first study evaluating effects of TNF-α treatment on DAPK1 and autophagy marker LC3B in first-trimester and term placental tissue. We show that placental DAPK1 protein is downregulated in response to TNF-α, irrespective of gestational age, whereas regulation of placental LC3B expression and its lipidation seem to differ between first-trimester and term of human pregnancy. Consistent with findings in TNF-α-treated term placental explants, we show that LC3B-I and II levels are increased in primary trophoblasts from early onset PE, while DAPK1 is decreased compared to controls. Based on these observations, it is tempting to speculate that elevated maternal TNF-α levels dysregulate autophagic machinery in human placenta through different routes depending on different stages of gestation.

The expression profiles of genes involved in autophagy seem to be largely overlapping in human first-trimester villous placenta in response to inflammatory cytokines IL-6 and TNF-α. While TNF-α elicits transcriptional regulation mainly via JNK (Kalliolias and Ivashkiv 2016), IL-6 receptor signaling is relayed to the nucleus via JAK/STAT signaling (Hunter and Jones 2015). However, both signaling cascades involve the NFκB and ERK pathways which could be the common factors leading to similar transcriptional responses in first-trimester placental explants. In this regard, both cytokines seem to have a similar regulatory role, with a much higher proportion of downregulated, compared to upregulated autophagy-related genes. Results from autophagy array and targeted qPCR analysis show good concordance for downregulation of placental DAPK1, whereas autophagy array data for CTSD and HDAC6 are only confirmed by trend. This may be explained by differences in experimental setup, including sample pooling for PCR array, differences in primer sequences and use of different reference genes. However, data from Western blotting and immunohistochemistry support gene expression data and overall suggest downregulation of placental DAPK1 in response to TNF-α in first trimester of pregnancy. This is in agreement with studies, suggesting DAPK1 inhibition on a transcriptional level by the pro-inflammatory transcription factors STAT3 and NFκB (Benderska and Schneider-Stock 2014), both of which are downstream players in the IL-6 and/or TNF-α signaling. DAPK1 plays key roles in various signaling processes including apoptosis and autophagy (Bialik and Kimchi 2006; Jin et al. 2006). One of its actions is phosphorylation of beclin 1, which then dissociates from its inhibiting anti-apoptotic protein Bcl-XL and associates with phosphatidylinositol 3-kinase catalytic subunit type 3 (PIK3C3) to induce autophagy (Zalckvar et al. 2009). Recently, DAPK1 has been suggested to contribute to resveratrol-induced autophagy in human dermal fibroblasts (Choi et al. 2013). In these cells, resveratrol treatment increased levels of LC3-II and GFP-LC3 punctate foci, reflecting its subcellular localization in autophagosomes, whereas DAPK1 silencing significantly attenuated observed effects. In agreement with that study, knockdown of DAPK1 in the hepatoma cell line HepG2 significantly decreased the LC3-II/I ratio in quinocetone-induced autophagy (Zhou et al. 2016). Together, these studies and our results in first-trimester placentas suggest that DAPK1 expression is directly associated with LC3-II levels in the autophagy process. However, our data from term placenta explants suggest that TNF-α-dependent regulation of the DAPK1 / LC3B axis may change during gestation. While at late stages of pregnancy DAPK1 still decreases in response to TNF-α, LC3B expression and lipidation in turn increase, suggesting uncoupling of placental DAPK1 levels from LC3B regulation at term of pregnancy.

Increased TNF-α is associated with a number of adverse pregnancy conditions including gestational hypertension and gestational diabetes mellitus (GDM) (Pantham et al. 2015; Peracoli et al. 2007). Since DAPK1 is upregulated in placentas from GDM pregnancies, our results suggest that rather the diabetic milieu or factors other than IL-6 and TNF-α regulate placental DAPK1 in diabetic pregnancies (Magee et al. 2014). At this point it should be stressed that besides TNF-α other inflammatory cytokines have been shown to induce DAPK1 expression in different cell models. Accordingly, transforming growth factor (TGF)-β treatment rapidly induced DAPK mRNA and protein expression in Hep3B hepatoma cells (Jang et al. 2002). Moreover, studies with mouse embryonic fibroblasts and bone marrow-derived macrophage cell lines suggest IFN-γ-induced DAPK1 upregulation through transcription factors C/EBP-β and ATF6 (Gade et al. 2008, 2012).

According to our array and qPCR data, first trimester placental LC3B (MAP1LC3B) mRNA expression seems to be unchanged in response to inflammatory cytokines IL-6 and TNF-α, which is in contrast to another study showing a significant reduction of LC3B expression in *chorion laeve* of pre-term delivery cases with signs of histological inflammation compared to cases without inflammatory lesions. This discrepancy may be explained by different gestational age of placenta samples, again suggesting gestational age-dependent changes in placental LC3B regulation in response to inflammatory cytokines. In our study, both first-trimester and term placenta predominantly shows the lipidated form of posttranslationally processed LC3B protein (LC3B-II). This is in line with previous studies in second trimester and term placenta (Oh et al. 2008), suggesting induction of autophagy at all stages of pregnancy. Of note is that a previous correlation analysis suggested decreasing LC3B-II levels with advancing gestational week (Akaishi et al. 2014). However, our observation of decreased LC3B-II in first-trimester placenta in response to TNF-α is in stark contrast to a previous study showing increased LC3B-II levels in TNF-α-treated Jeg-3 trophoblast cells (Oh et al. 2008). This discrepancy may be explained by the cancerous character of the choriocarcinoma cell line Jeg-3, whereas villous trophoblasts in placental explants remain in situ in their natural microenvironment. Additionally, Jeg-3 cells show a point mutation in one allele of the coding region of the p53 gene (Yaginuma et al. 1995). Since p53 is a regulator of autophagy and a direct substrate of DAPK1 (Bialik and Kimchi 2014; Feng et al. 2015), results in Jeg-3 cells may be explained by altered p53 downstream effects.

While a number of studies have demonstrated a role for TNF-α in stimulating autophagy in various cells, including macrophages, vascular smooth muscle, and skeletal muscle cells, its regulation is still not completely understood and may clearly differ between different cell types and tissues (Harris 2011) and developmental stages. For human placenta, autophagy is considered as crucial mechanism to adapt to maternal stress situations and disruption of this mechanism may contribute to placental dysfunction. We conclude that increased maternal TNF-α provokes a shift in the expression profile of genes involved in autophagy, which in first trimester of pregnancy is characterized by a higher proportion of downregulated than upregulated placental genes. Downregulation of placental DAPK1, a regulator of apoptosis, autophagy, and programmed necrosis, may consequently lead to an unbalanced crosstalk between these pathways, entailing different regulation routes of placental autophagy at different stages of human gestation.
